# Binding of Inhibitors to Nuclear Localization Signal Peptide from Venezuelan Equine Encephalitis Virus Capsid Protein Explored with All-Atom Replica Exchange Molecular Dynamics

**DOI:** 10.1021/acsomega.4c06981

**Published:** 2024-09-16

**Authors:** Bryan M. Delfing, Xavier E. Laracuente, Xingyu Luo, Audrey Olson, William Jeffries, Kenneth W. Foreman, Mikell Paige, Kylene Kehn-Hall, Christopher Lockhart, Dmitri K. Klimov

**Affiliations:** † School of Systems Biology, George Mason University, Manassas, Virginia 20110, United States; ‡ Department of Chemistry and Biochemistry, George Mason University, Fairfax, Virginia 22030, United States; § Center for Molecular Engineering, George Mason University, Manassas, Virginia 20110, United States; ∥ Department of Biomedical Sciences and Pathobiology, Virginia-Maryland College of Veterinary Medicine, Virginia Polytechnic Institute and State University, Blacksburg, Virginia 24061, United States; ⊥ Center for Emerging, Zoonotic, and Arthropod-borne Pathogens, Virginia Polytechnic Institute and State University, Blacksburg, Virginia 24061, United States

## Abstract

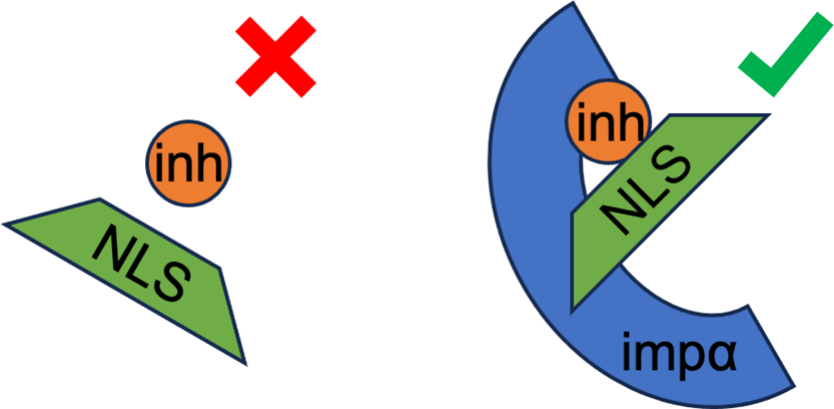

Several small molecule inhibitors have been designed to block binding of the Venezuelan equine encephalitis virus (VEEV) nuclear localization signal (NLS) sequence to the importin-α nuclear transport protein. To probe the inhibition mechanism on a molecular level, we used all-atom explicit water replica exchange molecular dynamics to study the binding of two inhibitors, I1 and I2, to the coreNLS peptide, representing the core fragment of the VEEV NLS sequence. Our objective was to evaluate the possibility of masking wherein binding of these inhibitors to the coreNLS occurs prior to its binding to importin-α. We found that the free energy of I1 and I2 binding to the coreNLS is less favorable than that to importin-α. This outcome argues against preemptive inhibitor binding to the coreNLS prior to importin-α. Instead, both inhibitors are expected to compete with the coreNLS peptide for binding to importin-α. The two factors responsible for the low affinities of the inhibitors to the coreNLS peptide are (i) the low cooperativity of binding to the peptide and (ii) the strong hydrophobic effect associated with binding to importin-α. Our results further show that upon binding to the coreNLS peptide, the inhibitors form multiple diverse binding poses. The coreNLS peptide coincubated with I1 and I2 adopts several conformational states, including open and collapsed, which underscores the fluidity of the coreNLS conformational ensemble as a target for inhibitors. Taken together with our prior investigations, this study sheds light on the molecular mechanism by which I1 and I2 ligands inhibit binding of the VEEV capsid protein to importin-α.

## 1 Introduction

**Figure 1:**
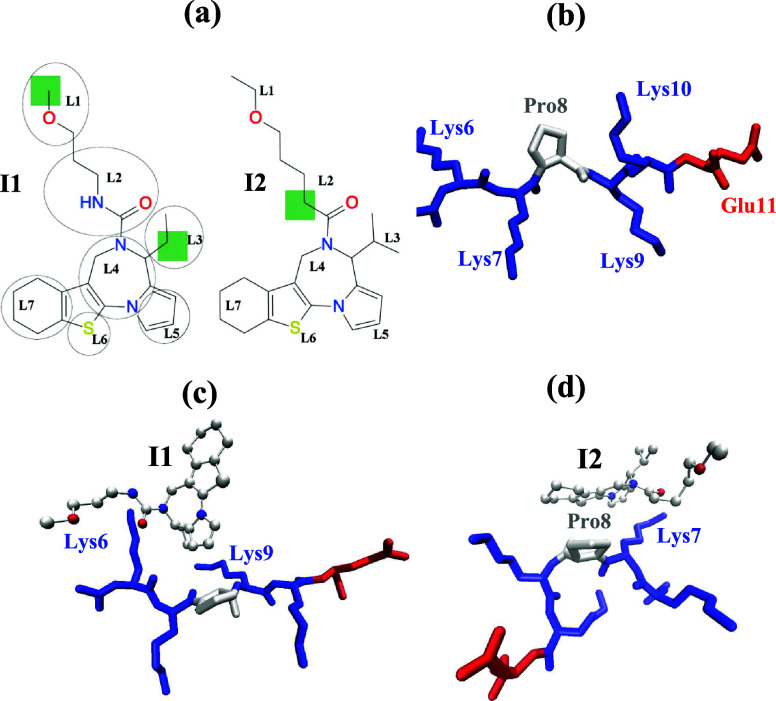
(a) Chemical structures of inhibitors G281-1564 (I1) and I2 with their structural groups identified. Differences in inhibitor structures are marked in green. (b) Structure of the coreNLS peptide KKPKKE. (c,d) Representative structures of the coreNLS peptide with bound I1 (c) and I2 (d) inhibitors. I1 primarily binds to the amino acids Lys6-Lys9, whereas I2 binds to Lys7 and Pro8 (see the text for details). Inhibitor apolar carbon and sulfur atoms are shown in light gray, whereas polar oxygen or nitrogen atoms are in red or blue. The coloring in parts (b–d) is amino acid-specific.

Venezuelan equine encephalitis virus (VEEV) is a highly infectious alphavirus capable of producing epidemic outbreaks, which is recognized for its potential use as a bioweapon.^1−3^ In humans, VEEV typically causes flu-like symptoms, although 14% of cases escalate into severe neurological disorders, including encephalitis, and some cases become fatal. As of now no FDA-approved vaccines or antivirals are available.^2^ Prior studies have shown that the molecular mechanism of VEEV’s infection involves its capsid protein binding to the nuclear import adaptor protein importin-α (impα) via its N-terminal nuclear localization signal (NLS) sequence.^4,5^ The resulting complex further recruits the nuclear export protein CRM1 and nuclear import partner protein importin-β, and as a tetramer, it blocks the nuclear pore complex (NPC) interfering thus with nucleocytoplasmic traffic.^2,5^ Because this NPC-clogging complex plays a key role in the disease mechanism, one may expect that inhibiting the binding of the VEEV NLS sequence to impα is a viable therapeutic option.^5−7^ Implementing this strategy, several small compounds from the CL6662 scaffold family prepared for the Queensland Compound Library Open Scaffolds collection^8^ have been considered as inhibitors.^9^ Among these compounds was G281-1564 (designated as I1 in Figure 1a), which was selected based on its demonstrated inhibition of the VEEV infection and specificity toward VEEV’s NLS compared to the simian virus SV40 large T-antigen NLS.^9^ In our previous studies, we also introduced a hydrophobic version of I1 termed I2 (Figure 1a). We have analyzed binding of these ligands to impα in the absence of the viral NLS and showed that I1 and I2 bind to the impα major NLS binding site diffusively without forming specific poses.^10^

To be effective, VEEV inhibitors may need to compete for binding to impα the VEEV NLS sequence. Its impα binding sequence A_5_KKPKKE_11_ spans the amino acid positions P1–P7 and follows a classic monopartite NLS consensus sequence K-K/R-X-K/R putting Lys, Lys, Pro, and Lys at P2–P5.^11^ This NLS sequence constitutes a part of a longer capsid fragment bound to a mouse impα2 protein with its structure being resolved experimentally (PDB entry 3VE6(12)). Our recent molecular dynamics simulations have investigated the mechanism of VEEV NLS binding to impα.^13^ We found that upon binding to impα the 6-mer NLS sequence, K_6_KPKKE_11_, termed the coreNLS, adopts a native pose seen in 3VE6, while the shorter sequences, K_6_KPK_9_ or K_6_KPKK_10_, failed to reproduce the crystallographic native binding structure.^13^ We suggested that the coreNLS sequence is sufficient but not necessary for native binding. This conclusion stems from the observation that there is at least one other sequence KKPKIR (PDB 4WV6) conferring the same bound pose as VEEV’s coreNLS does. It is of note that in the native binding pose, the coreNLS forms multiple strong binding interactions with impα. Specifically, Lys6 makes an electrostatic contact with Asp122 of impα, while Lys7 and Lys9 occupy the cages formed by impα Trp72, Trp114, and Trp161 and establish π–cation interactions with their indole rings.

In our previous studies, we used the experimental EC50 values for CL6662 inhibitors to estimate their free energy of binding to impα Δ*G*_b_, which was found to be around −7 kcal/mol.^10^ It has been argued in the literature that a typical binding free energy for an NLS sequence, e.g., from SV40 virus, is about −10 kcal/mol.^14^ More recent estimates for some NLS sequences from, e.g., chloride intracellular channel proteins, have reduced Δ*G*_b_ to about −5 kcal/mol.^15^ Since VEEV and host NLS sequences are similar, their Δ*G*_b_ values should also be in the same range. If so, the free energies of VEEV NLS and inhibitor binding are comparable, and the binding affinities of inhibitors might be sufficient to disrupt, if not block, the VEEV NLS binding. To directly test this hypothesis, we performed all-atom replica exchange molecular dynamics simulations probing the competitive binding of the VEEV coreNLS peptide and I1 or I2 ligand to the impα major NLS binding site.^16^ Strikingly, we found that both inhibitors completely abrogate the native binding of the coreNLS peptide, which is forced to adopt a manifold of non-native loosely bound poses. We also determined that when bound to impα, both inhibitors destabilize the native coreNLS binding by masking its amino acids rather than by interfering with the binding interactions formed by impα. We showed that I1 is more effective in inhibiting the coreNLS native interactions than I2, and that conclusion was confirmed by AlphaScreen experiments measuring the IC50 values for both inhibitors.

However, our previous studies have left one important question unanswered: could the inhibitors mask the VEEV NLS prior to its binding to impα? The feasibility of such a masking scenario is supported by multiple experimental studies.^17^ For example, intermolecular masking has been observed for the transcription factor NF-κB,^18^ caspase-2, or FOXO forkhead transcription factor proteins.^19^ In all of these cases, an external molecule shields or “masks” the NLS sequence, preventing its binding to impα. Typically, this process is highly dynamic allowing a cell to carefully regulate intranuclear localization of these proteins. To determine whether inhibitors I1 and I2 can utilize this intermolecular masking mechanism, we used all-atom explicit water replica exchange molecular dynamics to study their binding to the coreNLS peptide in the absence of impα. Our central result is that I1 and I2 binding to the coreNLS is unfavorable compared to the binding to impα. Furthermore, we identify factors responsible for the low affinity of the inhibitors to the coreNLS peptide. We also show that upon binding to the coreNLS, neither I1 nor I2 adopts specific binding poses, sampling instead multiple diverse positions around the peptide. Taken together with our prior investigations, this study allowed us to answer the question about masking feasibility and map the molecular inhibition mechanism.

## 2 Results and Discussion

### 2.1 Binding Free Energies of VEEV Inhibitors

The coreNLS peptide KKPKKE represents a nuclear localization signal (NLS) sequence fragment from the VEEV capsid protein. According to the PDB structure 3VE6, this peptide fragment tightly binds to the importin-α (impα) transport protein, forming multiple interactions, including salt bridges, π–cation interactions, and hydrogen bonds. We showed in our previous publication^16^ that the inhibitor G281-1564 (I1) and its more hydrophobic variant I2 completely abrogate native binding of the coreNLS peptide to the impα major NLS binding site. As a result of their interference, the coreNLS is forced to adopt a manifold of non-native loosely bound poses. Importantly, when bound to impα, the inhibitory action of these ligands primarily results from destabilizing the native binding interactions formed by the coreNLS, i.e., effectively from masking its amino acids, rather than from targeting impα interactions with the peptide. This conclusion raises the possibility that these inhibitors may bind the VEEV capsid NLS sequence even before its binding to impα resulting in “preemptive” NLS masking. To investigate this scenario, we performed all-atom REST simulations probing the binding of I1 and I2 inhibitors to the coreNLS peptide in water in the absence of impα. To provide a reference, we performed the REST simulations of the coreNLS peptide in a ligand-free water. Below, we present their analysis and compare them with the binding of the same inhibitors to impα studied previously.^10^

Our new simulations show that at 310 K, the inhibitor I1 binds to the coreNLS peptide with the probability *P*_b_ = 0.65 ± 0.02. The I2 ligand shows a lower binding affinity of *P*_b_ = 0.56 ± 0.03. Using these probabilities, we estimate the free energy of inhibitor binding to the peptide by applying the expression
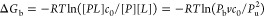
1where [*PL*], [*P*], and [*L*] are the concentrations of the coreNLS + inhibitor complex, coreNLS peptide, and inhibitor, respectively, while *c*_0_ is the standard concentration. We further assume that [*PL*] = *P*_b_/*v*, [*P*] = *P*_u_/*v*, and [*L*] = *P*_u_/*v*, where *v* is the volume of the sphere restraining the peptide and the ligand (see Section 4), *P*_u_ is the probability of ligand unbinding, and binding is two-state, i.e., *P*_b_ + *P*_u_ = 1. Because this computation of Δ*G*_b_ postulates no interactions between a ligand and a peptide in the unbound state,^20^ we verify this assumption in SI. Then, for I1, we get Δ*G*_b_ ≈ −2.4 kcal/mol, which is reduced to −2.0 kcal/mol for I2. Thus, the I2 inhibitor has a lower binding affinity to the coreNLS than I1. Given a highly polar state of the coreNLS peptide and the fact that I1 is more hydrophilic than I2, the stronger affinity of the former is expected. Because binding free energy may depend on the definition of a bound state,^21^ we tested its alternative definitions in the SI. The results in Table S1 indicate that Δ*G*_b_ values depend weakly on the specific bound state definition and do not alter our conclusions.

To get further insights, we compare the affinities of the inhibitors to the coreNLS peptide and the impα major NLS binding site. Using the data from our previous study^10^ and its analysis in the SI, we found that the probabilities of I1 and I2 binding to impα are *P*_b_ = 0.96 ± 0.02 and 0.97 ± 0.01, respectively. Taking into account that in the previous and current simulations the volume available for a ligand is about the same and using eq 1, we estimate Δ*G*_b_ for binding to impα to be −5.2 and −5.5 kcal/mol for I1 and I2, respectively. It is of interest to relate these Δ*G*_b_ values to the experiments. Although we are not aware of experimental studies probing the interactions of the inhibitors I1 and I2 with the coreNLS peptide, the inhibitory activity of I1 blocking interactions between impα and VEEV capsid has been measured.^9^ Indeed, if we neglect the contribution of interligand interactions to binding, we can apply Clark’s equation^22^ that assumes EC_50_ ≈ *K*_D_, where *K*_D_ is an inhibitor dissociation constant, and EC_50_ is a half-maximum effective concentration. Since Δ*G*_b_ = −*RT*ln(*c*_0_/*K*_D_), and I1 EC_50_ = 10.8 ± 4.3 μM,^9^ we find Δ*G*_b_ = −6.8 ± 0.2 kcal/mol. Given that the simulations are inherently reductionist (see SI comment on binding state definition), we believe that our estimate of Δ*G*_b_ ≈ – 5.2 kcal/mol is reasonably consistent with the experimental measurement. It is also worth noting that qualitatively similar results follow from our previous analysis of binding affinities using AutoDock.^16^ There we found that the free energies of I1 and I2 binding to the coreNLS peptide are −4.0 ± 0.0 and −3.9 ± 0.1 kcal/mol, respectively, which are higher than the corresponding AutoDock affinities to impα of −6.0 ± 0.1 and −6.2 ± 0.0 kcal/mol. Thus, independent of the precise affinity estimate based on REST simulations or AutoDock, our analysis unambiguously shows that both inhibitors exhibit a thermodynamic preference for binding to the impα protein rather than to the coreNLS peptide. This is a central conclusion of our study, which we substantiate below.

### 2.2 The Mechanism of Inhibitors’ Binding to the coreNLS Peptide

We next analyze the binding interactions formed between the inhibitors and the coreNLS peptide. Consistent with I1 stronger binding affinity, there are ⟨*C*⟩ = 2.0 ± 0.1 binding contacts between I1 and the coreNLS, whereas for I2, ⟨*C*⟩ = 1.6 ± 0.1. It is important to examine the cooperativity of the inhibitor binding. When bound, I1 or I2 simultaneously interact with ⟨*C*_b_⟩ = 3.1 ± 0.1 or 2.9 ± 0.1 coreNLS amino acids. Thus, upon binding, both inhibitors engage about half of the coreNLS peptide. Furthermore, we can directly compute the probability *P*_s_ that upon binding, an inhibitor interacts with a single coreNLS amino acid. We found that such probabilities are low being 0.26 ± 0.01 for I1 and 0.31 ± 0.00 for I2. Hence, bound inhibitors typically interact with multiple coreNLS amino acids. However, when similar computations are performed for binding to impα, we discover that these ligands interact simultaneously with ⟨*C*_b_⟩ = 8.6 ± 0.4 and 9.5 ± 0.8 impα amino acids. Because the coreNLS binding site in the 3VE6 structure is composed of 21 amino acids,^13^ I1 or I2 interact with 41 or 45% of those amino acids. Moreover, the probability for I1 or I2 to interact with a single impα amino acid upon binding is vanishingly small being *P*_s_ ≈ 0.01 for both. Thus, the comparison of inhibitor binding to the coreNLS and impα argues that the latter is far more cooperative than the former. In fact, if we measure the binding cooperativity by ⟨*C*_b_⟩, then binding to impα is 3-fold more cooperative than to the coreNLS. It is worth noting that the low cooperativity in binding to the coreNLS is not due to the size of the peptide because the ligand engages only about half of coreNLS amino acids. We believe that the root cause is the entropic costs stemming from organizing coreNLS amino acids around the inhibitor. Indeed, the low cooperativity of the inhibitor binding to the coreNLS might be expected, if we take into account that impα is a folded protein with a well-structured major NLS binding site, whereas the coreNLS is unstructured.^2^ More importantly, this analysis suggests a factor limiting the binding affinity of the inhibitors to the coreNLS peptide that is weak binding cooperativity compared to that observed upon their binding to impα.

**Figure 2:**
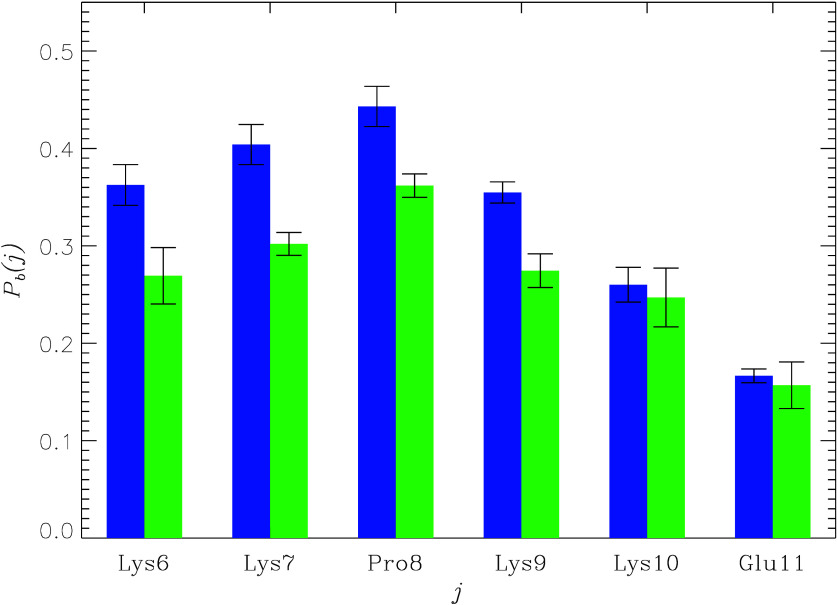
Probabilities *P*_b_(*j*) of inhibitor binding to the coreNLS amino acids *j*. The blue and green data are collected for I1 and I2 binding. The figure shows that for all amino acids, the binding affinity of I1 is stronger than that of I2.

Figure 2 presents the probabilities *P*_b_(*j*) of inhibitors binding to the coreNLS amino acids *j*. For both inhibitors, *P*_b_(*j*) peaks at *j* = Pro8 and sharply declines toward the peptide C-terminus. It also follows that I1 binds to each coreNLS amino acid *j* with a higher probability *P*_b_(*j*) than I2 with the largest differences observed at the positions *j* = Lys6 through Lys9. Figure S7 in SI shows the probabilities *P*_b_(*k*) of peptide binding to the ligand group *k* in both inhibitors. These plots indicate that the largest binding gains favoring I1 occur for ligand groups *k* = L2 and L4. The finding can be explained if we notice that in Figure 1a, these groups surround the I1 region with reduced hydrophobicity compared to I2. If so, then these differences in I1 and I2 binding may be due to hydrogen bonding between an inhibitor and the peptide. We found that, on an average, the number of ligand–peptide hydrogen bonds is small being ⟨*N*_HB_⟩ = 0.12 ± 0.01 for I1 and 0.06 ± 0.00 for I2. However, our analysis does show that the NH group present in I1 and deleted in I2 (see Figure 1a) accounts for about 30% of hydrogen bonds established by I1 with the peptide, suggesting the source of observed differences in ligand group affinities.

We next analyze the losses in solvent accessible surface areas (SASA) occurring upon binding of the inhibitor to the coreNLS. To this end, we compared the SASA of the complex and those of the coreNLS peptide and inhibitors when separated. Their changes are summarized in Table 1. It is seen that, consistent with the stronger affinity of I1, its SASA loss upon binding to the coreNLS peptide Δ*SASA* is almost 10% larger than that for I2. Interestingly, for both inhibitors, the loss in their apolar SASA Δ*hSASA* represents about 90% of their total Δ*SASA*. For comparison, this fraction is reduced to 68% for the coreNLS peptide. These calculations demonstrate that binding of both inhibitors, especially I2, engages almost exclusively their apolar atoms with little contribution from polar groups. It is instructive to examine the losses in SASA occurring upon binding of the inhibitors to impα, as presented in Table 2. (This new analysis is conducted using our previous data^10^). In this case, the losses in inhibitor apolar SASA also constitute more than 90% of the total inhibitor ΔSASA. More importantly, compared to the coreNLS, the binding of I1 to impα shields from water an additional apolar area of ΔΔ*h*SASA = −147 ± 14 Å^2^. For comparison, an additional burial of polar surface is almost 3-fold smaller being ΔΔ*p*SASA = −59 ± 6 Å^2^. For I2, the same quantities are −184 ± 19 and −70 ± 7 Å^2^. These calculations argue that binding to impα provides strikingly larger burial of the hydrophobic surface than binding to the coreNLS peptide, implicating the hydrophobic effect as a major factor driving binding. This conclusion is further bolstered by a small contribution of hydrogen bonding to the inhibitor binding to impα and the analysis of impα amino acids involved in binding.^10^ According to our previous study,^10^ among the top 10 impα amino acids binding I1 and I2, there are tryptophan amino acids Trp72, Trp114, and Trp161 and hydrophobic amino acids Leu34 and Ile42. These amino acids present a binding interface unavailable in the coreNLS. Therefore, the analysis of binding presented in this section identified two key factors contributing to weaker affinities of I1 and I2 to the coreNLS peptide compared to impα—(i) a low cooperativity of inhibitor binding to the peptide and (ii) a stronger hydrophobic effect occurring upon inhibitor binding to impα.

**Table 1:** Changes in Solvent Accessible Surface Areas upon Inhibitor Binding to the coreNLS Peptide^a^^,^^b^

simulation	ΔSASA, Å^2^	Δ*h*SASA, Å^2^	Δ*p*SASA, Å^2^
coreNLS + inhibitor			
I1	–212 ± 3	–166 ± 2	–46 ± 1
I2	–196 ± 1	–158 ± 1	–38 ± 1
peptide only^c^			
I1	–107 ± 1	–73 ± 1	–34 ± 0
I2	–99 ± 1	–67 ± 0	–33 ± 1
inhibitor only^d^			
I1	–105 ± 1	–93 ± 1	–12 ± 1
I2	–98 ± 0	–91 ± 1	–5 ± 0

a ΔSASA = SASA(*PL*) – SASA(*P*) – SASA(*L*), where SASA(*x*) refers to the SASA values of the complex (*x* = *PL*), peptide (*x* = *P*), or inhibitor (*x* = *L*).

b Δ*h*SASA and Δ*p*SASA are apolar and polar components of SASA.

c Data refer to the changes in the peptide SASA only.

d Data refer to the changes in the inhibitor SASA only.

**Table 2:** Changes in Solvent Accessible Surface Areas upon Inhibitor Binding to impα^a^^,^^b^^,^^c^

simulation	ΔSASA, Å^2^	Δ*h*SASA, Å^2^	Δ*p*SASA, Å^2^
impα + inhibitor			
I1	–419 ± 15	–313 ± 11	–105 ± 5
I2	–450 ± 24	–342 ± 18	–108 ± 6
impα only^d^			
I1	–201 ± 7	–110 ± 4	–90 ± 5
I2	–216 ± 10	–114 ± 3	–102 ± 7
inhibitor only^e^			
I1	–218 ± 8	–203 ± 8	–15 ± 0
I2	–234 ± 14	–228 ± 14	–7 ± 1

a ΔSASA = SASA(*PL*) – SASA(*P*) – SASA(*L*), where SASA(*x*) refers to the SASA values of the complex (*x* = *PL*), impα (*x* = *P*), or inhibitor (*x* = *L*).

b Δ*h*SASA and Δ*p*SASA are apolar and polar components of SASA.

c New analysis of the data from ref (10).

d Data refer to the changes in impα SASA only.

e Data refer to the changes in the inhibitor SASA only.

### 2.3 Conformational Ensembles of the Inhibitor-coreNLS Complex

**Figure 3:**
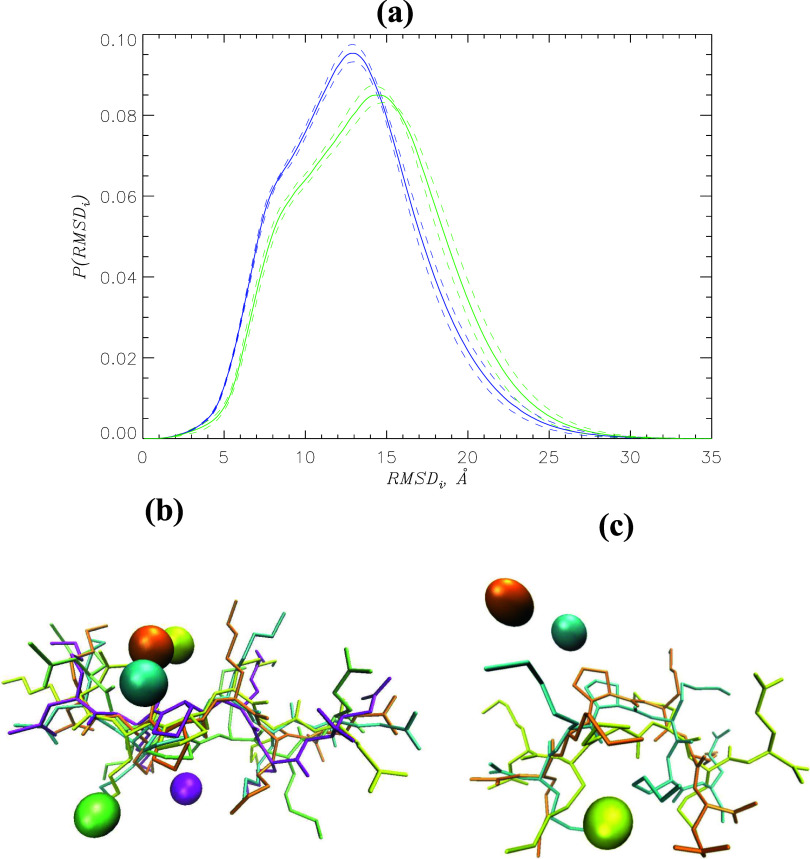
(a) Probability distributions *P*(RMSD_*i*_) of the bound ligand RMSD_i_ values computed after aligning the coreNLS peptide structures. Data in blue and green are collected for I1 and I2 binding, respectively. The error bounds are shown by dashed lines. (b,c) Centroids of the most populated bound clusters for I1 (b) and I2 (c) inhibitors shown around the coreNLS peptide. An inhibitor pose is represented by its center of mass as a sphere. The fractions of ligand poses retained by these clusters are low, being 0.034, 0.024, 0.022, 0.019, and 0.013 for I1 and 0.038, 0.035, and 0.014 for I2 (see Section 4). The peptide structures and inhibitor poses are colored in the descending order of cluster population, as orange, yellow, cyan, purple (I1 only), and lime (I1 only). The figure implicates scattered positions of the ligands around the peptide.

Do the inhibitors adopt specific poses upon binding to the coreNLS peptide? To answer this question, we use the probability distributions *P*(RMSD_*i*_) of the bound ligand RMSD_*i*_ values computed after aligning the coreNLS peptides. These distributions result from an all-against-all comparison of the inhibitor poses around the peptide. Figure 3a shows that for I1 and I2, *P*(RMSD_*i*_) are unimodal peaking at RMSD_*i*_ ∼ 15 Å and extending beyond 20 Å. In fact, the average RMSD_*i*_ values for the I1 and I2 bound poses are strikingly high being ⟨RMSD_*i*_⟩ = 12.9 ± 0.2 and 14.0 ± 0.1 Å, respectively. To illustrate the diversity of binding poses, we aligned the peptide conformations and then clustered the inhibitor poses around the coreNLS (see Section 4). Because none of the clusters capture more than 4% of bound ligands, the poses of several most populated clusters’ centroids are shown in Figure 3b,c. Then, Figure 3 and the dispersed distribution of ligands over clusters unequivocally demonstrate that neither I1 nor I2 adopts specific poses upon binding to the coreNLS occurring instead in diverse positions around the peptide.

**Figure 4:**
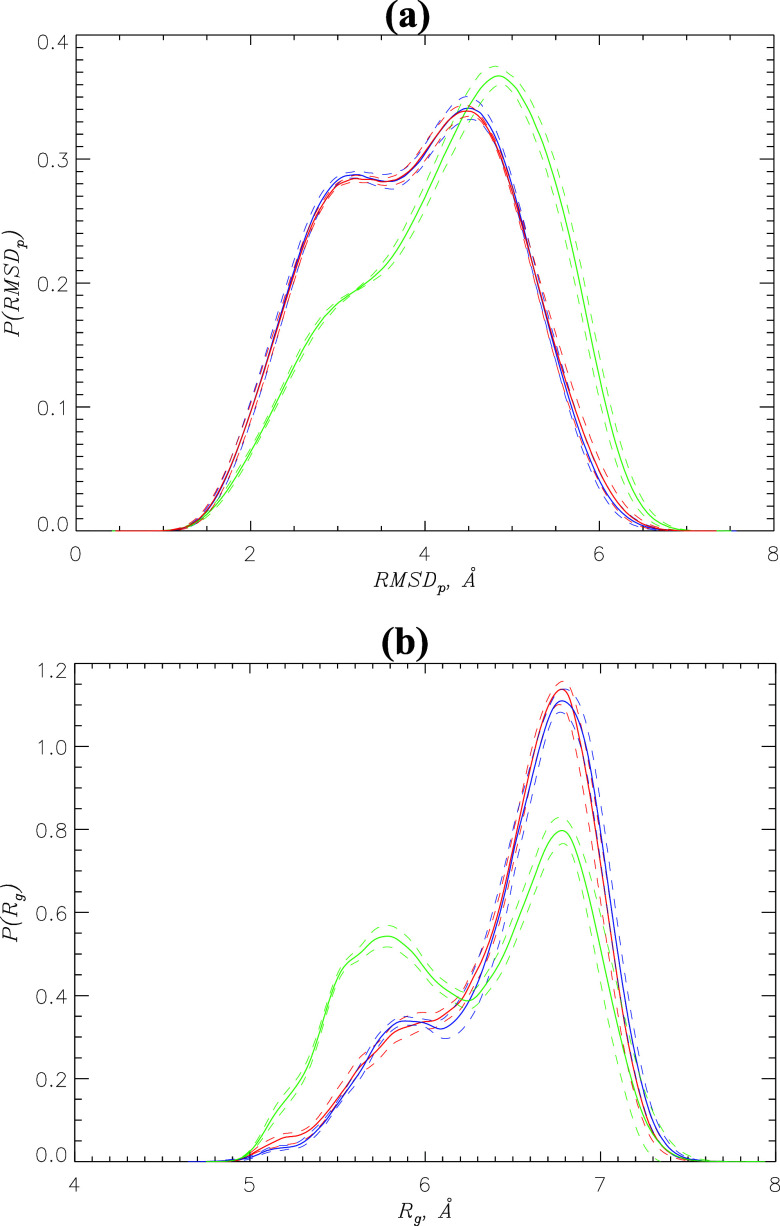
(a) Probability distributions *P*(RMSD_*p*_) of the peptides RMSD_p_ values computed after their alignment. (b) Probability distributions *P*(*R*_g_) of the coreNLS radius of gyration *R*_g_. In both panels, data in blue and green correspond to the peptide coincubated with I1 and I2, respectively. The data in red represent the ligand-free coreNLS peptide. The error bounds are shown by dashed lines. The figure suggests that binding of I2 but not I1 modifies the coreNLS structure.

The next question pertains to the possibility that the inhibitor changes the structure of the coreNLS peptide. Figure 4a shows the probability distributions *P*(RMSD_p_) of the peptide RMSD_p_ values computed after peptide alignment. These RMSD values result from all-against-all comparison of the coreNLS structures. The figure reveals distinct *P*(RMSD_p_), suggesting that the inhibitor does affect peptide structure. Indeed, the average RMSD_p_ values for I1 and I2 are 3.9 ± 0.0 and 4.3 ± 0.0 Å, respectively, indicating that the coreNLS ensemble coincubated with I2 is more diverse than with I1. Further evidence comes from the analysis of the probability distribution *P*(*R*_g_) of the peptide radius of gyration *R*_g_. Figure 4b reveals bimodal *P*(*R*_g_) observed upon I1 and I2 binding, indicative of two peptide conformational states, open and collapsed. We verified this conclusion by clustering the peptide conformations coincubated with I1 and I2 (see Section 4). For I1, we found two populated conformational clusters CL1 and CL2 with the respective populations of 0.71 ± 0.01 and 0.17 ± 0.01. The RMSD between the CL1 and CL2 centroids is 4.1 Å, suggesting that they represent distinct peptide conformations. CL1 features more open coreNLS structures with *R*_g_ ≈ 6.7 Å, whereas CL2 conformations are collapsed with a smaller *R*_g_ ≈ 6.0 Å. The overall peptide dimensions are characterized by the radius of gyration of 6.5 ± 0.0 Å. Clustering of the peptide ensemble coincubated with I2 also produced two populated clusters CL1 and CL2 with the RMSD between them of 5.0 Å and the populations of 0.49 ± 0.01 and 0.27 ± 0.02. Similar to the I1 ensemble, the cluster CL1 is open with the radius of gyration of *R*_g_ ≈ 6.8 Å, whereas CL2 captures collapsed peptide species with *R*_g_ ≈ 5.8 Å. The overall radius of gyration of the coreNLS peptide observed upon I2 binding is reduced to 6.3 ± 0.0 Å. Thus, although the peptide conformational ensembles coincubated with I1 and I2 exhibit qualitatively similar distributions, I2 binding causes a partial peptide collapse. This outcome can be explained by stronger hydrophobicity of I2, which upon binding to the coreNLS creates an apolar environment favoring the partial peptide collapse. Incidentally, the partial coreNLS collapse is illustrated by the “yellow” cluster, as shown in Figure 3c. More generally, the existence of multiple coreNLS conformational states upon coincubation with I1 and I2 underscores the structural fluidity of the coreNLS-inhibitor complex and the lack of a specific peptide structure that can be targeted by ligands.

It is instructive to compare the coreNLS conformational ensembles coincubated with I1 and I2 with that sampled in a ligand-free water. Surprisingly, Figure 4 reveals that binding of I1 produces no impact on the coreNLS conformations, whereas I2 forces a partial peptide collapse. Indeed, the average coreNLS radius of gyration ⟨*R*_g_⟩ in a ligand-free water is 6.5 ± 0.0 Å, which is identical to that for I1 binding. Furthermore, clustering of the ligand-free peptide structures results in two populated clusters, CL1 and CL2, with populations of 0.69 ± 0.01 and 0.17 ± 0.01, respectively. The RMSD between the centroids of CL1 clusters in a ligand-free water and upon I1 binding is 2.0 Å, whereas for CL2, it is 2.2 Å. If we select the coreNLS ensemble coincubated with I2 and perform the same comparisons, then the respective RMSDs are 3.8 and 3.3 Å. Thus, the coreNLS conformational ensembles in a ligand-free water and coincubated with I1 are similar, whereas I2 alters the peptide structures. These observations imply that interactions of I1 with the coreNLS peptide effectively resemble water hydration. The conclusion is unexpected given obvious differences in the structures of I1 and water. However, we note that I1 molecule has a hydrogen bond donor in L2 and several acceptors in L1 and L2 (Figure 1a). In contrast, with no donor groups, I2 can only accept hydrogen bonds, and due to two extra methyl groups, it is more hydrophobic than I1. Taken together, these factors are apparently responsible for I2 binding changing the coreNLS structure, whereas I1 leaves it intact.

### 2.4 Broader Implications

Intramolecular masking of the NLS sequence by conformational changes that bury the NLS is well-documented in the literature.^17^ For example, in a phosphorylated form, the nuclear factor of activated T-cells NF-AT4 folds preventing the access to the NLS region, whereas dephosphorylation causes the exposure of its NLS.^23^ Another example comes from cancer cell-specific proapoptotic factor viral protein 3, whose binding to the impα/β heterodimer is regulated by its conformation.^24^ Truncation of 73 N-terminal amino acids unmasks the NLS promoting binding to impα/β. Similarly, the N-terminal NLS sequence in a full-length non-DNA binding precursor NF-κB p110 is not accessible but becomes activated with the removal of C-terminal amino acids.^25^ This observation suggests that the C-terminus shields the NLS region in this protein. An example more closely related to our study is given by the cryptic NLS region in zinc finger RNA binding protein dTIS11.^26^ As long as the zinc finger region of dTIS11 coordinates zinc ion, the NLS is hidden; however, the mutations disrupting zinc binding unmask the NLS and facilitate nuclear import. These examples prompt us to expect that if inhibitor binding to the coreNLS results in its conformational change, masking may be promoted. Indeed, this scenario would reduce the free energy of coreNLS binding to impα Δ*G*_b_. Our findings suggest that this factor is irrelevant to the ability of our inhibitors to “mask” the coreNLS peptide prior to its binding to impα. Whether an inhibitor alters the coreNLS structure or not, the weak binding affinity of the inhibitor to the coreNLS peptide cannot outcompete a much more favorable binding to impα. Nevertheless, we cannot rule out that the fragments of the NLS sequence from the VEEV capsid longer than the coreNLS may still utilize intramolecular masking mechanisms.

In addition, there are multiple cases of intermolecular masking of NLS sequences.^17^ An example is the regulation of the nuclear import of the transcription factor NF-κB involved in tumorigenesis, immune and inflammatory responses.^18^ Inhibitor protein IκB binds NF-κB, masks its NLS, and blocks its nuclear import. Phosphorylation of IκB makes the NLS accessible and the nuclear import ready. Furthermore, scaffolding 14–3–3 protein shields the NLS regions in caspase-2 and FOXO forkhead transcription factor proteins, preventing their nuclear import.^19^ In this context, it is theoretically feasible that I1 and I2 could mask the VEEV coreNLS peptide before binding to impα. However, our findings rule this possibility out due to the much stronger binding affinity of the inhibitors to impα than to the coreNLS. As a result, the primary inhibition mode for both I1 and I2 involves their competitive binding with the coreNLS to impα. Effectively, in this scenario, impα offers itself as a template for such binding competition. In the previous paper, we showed that both inhibitors competing with the coreNLS for binding to impα completely abrogate the native binding of the peptide.^16^ Interestingly, both inhibitors destabilize the native coreNLS binding by targeting its amino acid binding interactions rather than those formed by impα amino acids. In this sense, the inhibiting action of I1 or I2 can be described as “masking” the coreNLS peptide bound to impα. Importantly, the current study shows that such “masking” can occur only when the coreNLS and inhibitor are *already bound* to impα ruling out the preemptive “masking” prior to binding to impα. Such an inhibition scenario raises a question about the selectivity of I1 and I2 inhibitors against VEEV NLS. Prior experimental studies offered some support for I1 selectivity,^9^ and according to our analysis, the coreNLS sequence is found in only one (!) human protein interacting with impα.^13^ Nevertheless, it is important to test the I1 and I2 selectivity through direct REST simulations, probing their competition with other NLS sequences for binding to impα.

## 3 Conclusions

We used all-atom explicit water REST simulations to study the binding of two inhibitors, I1 and I2, to the coreNLS peptide, representing the core fragment of the NLS sequence from the VEEV capsid protein. Our objective was to evaluate the possibility of masking, i.e., binding of these inhibitors to the coreNLS prior to its binding to impα transport protein. To this end, we computed the free energies of inhibitors binding to the coreNLS peptide and impα and evaluated the binding mechanisms. Succinctly, our conclusions can be summarized as follows. First, the inhibitor binding to impα is thermodynamically more favorable than binding to the coreNLS peptide. This outcome rules out the scenario wherein an inhibitor preemptively masks the coreNLS peptide before its binding to impα. Instead, both inhibitors are expected to compete with the coreNLS peptide for binding to the impα major NLS binding site. Second, we identified two likely factors responsible for the low affinities of the inhibitors toward the coreNLS peptide. These are (i) the low cooperativity of inhibitor binding to the peptide compared to the binding to impα and (ii) a stronger hydrophobic effect associated with inhibitor binding to impα. Third, our results show that upon binding to the coreNLS peptide, the inhibitors do not form any specific binding poses adopting instead a multitude of diverse bound positions around the peptide. Fourth, the coreNLS peptide coincubated with I1 and I2 adopts several conformational states, including open and collapsed, which underscores the fluidity of the coreNLS conformational ensemble as a target for inhibitors. Taken together with our prior investigations, this study sheds light on the molecular mechanism by which I1 and I2 ligands inhibit binding of the VEEV capsid protein to impα transport protein.

## 4 Models and Methods

### 4.1 Simulation System

We considered two simulation systems, each including one inhibitor and one coreNLS peptide K_6_KPKKE_11_ from VEEV’s capsid NLS sequence (see Figure 1).^5^ The two inhibitors analyzed were G281-1564 (I1) and its hydrophobic variant I2. I1 has been studied experimentally,^9^ whereas binding of both to impα has been investigated in our previous simulations.^10,27^ Following our previous studies, neutral acetylated and amidated groups were added to the peptide termini. The centers of mass of the peptide and ligand were restrained to the sphere with a radius of 15.75 Å using soft harmonic potential. The sphere center was fixed. Its radius was selected to match the protein-free volume in our previous simulations, which studied binding of I1 and I2 to impα.^10^ This system setup maintains the same effective concentration of inhibitors upon binding to impα or to the coreNLS peptide, permitting direct comparisons of inhibitor affinities.

The all-atom CHARMM36m force field^28^ and the CHARMM General Force field (CGenFF)^29,30^ were used to model the coreNLS and inhibitors, respectively. Water molecules were modeled using the CHARMM-modified TIP3P model.^31,32^ Both systems were solvated in a cubic water box with a side length of about 42.5 Å. Ten chloride ions and seven sodium ions were added to the systems to produce a salt concentration of approximately 150 mM while neutralizing the overall system charge. The I1 and I2 systems have similar numbers of water molecules, 2490 and 2495, respectively. The total numbers of atoms in each system were 7669 and 7691. For reference, we also studied the ligand-free simulation system, which, except for the absent inhibitors, was designed in the same way as the I1 or I2 systems. Together, these three simulation systems were used to probe the binding of I1 or I2 to the coreNLS fragment from VEEV’s capsid protein and compare it to the binding of the same ligands to impα (Figure 1).

### 4.2 Replica Exchange Simulations

We utilized isobaric–isothermal replica exchange with solute tempering (REST) molecular dynamics^33^ to sample the binding of ligands to the peptide. Because the full REST documentation is available elsewhere,^33,34^ we provide its brief summary below. We used *R* = 10 REST temperature conditions, which followed the geometrical distribution between *T*_0_ = 310 K and *T*_*R*–1_ = 510 K. For ligand-free simulations, we used *R* = 8 REST temperature conditions. Replicas *r* and *r* + 1, residing at the adjacent temperatures *m* and *m* + 1, exchange at a rate of ω = min[1, *e*^–Δ^], where Δ = β_*m*_(*H*_*m*_(*X*_*r*+1_) – *H*_*m*_(*X*_*r*_)) + β_*m*+1_(*H*_*m*+1_(*X*_*r*_) – *H*_*m*+1_(*X*_*r*+1_)), β = (*R*_c_*T*)^−1^, *H* is the enthalpy, *X* refers to system coordinates, and *R*_c_ is the gas constant. Solvent–solvent and solute–solvent interactions at a given temperature *T*_m_ were scaled by the factors *T*_m_/*T*_0_ and (*T*_m_/*T*_0_)^1/2^, respectively. This scaling strengthens the solvent–solvent interactions and excludes them from ω, reducing therefore the number of replicas required with no impact on the temperature range or exchange rates. The coreNLS and inhibitors were fully tempered, while water and ions were left effectively “cold” without tempering. Exchanges between replicas were attempted at 2 ps intervals, succeeding roughly with the exchange probability of 0.39 for each system.

The REST simulations were performed with the program NAMD,^35^ using periodic boundary conditions and a 1 fs integration step. Hydrogen-based covalent bonds were constrained via the SHAKE algorithm. Electrostatic interactions were computed through Ewald summations, and van der Waals interactions were smoothly switched off from 8 to 12 Å. The temperature was controlled using underdamped Langevin dynamics with a damping coefficient of γ = 5 ps^–1^. The system pressure was set to 1 atm through the Nosé–Hoover Langevin piston method using a piston period of 200 fs and a decay of 100 fs. The *x*, *y*, and *z* dimensions of each system were coupled.

Four REST trajectories were produced for each system. The initial structures for each REST trajectory were prepared in the following manner. The coordinates of the peptide and ligand, if present, were taken from the initial structures of the production REST simulations in our previous competitive binding study,^16^ then placed in the restraining sphere, solvated, and ionized. These structures underwent minimization, heating to 310 K, and subsequent 1 ns equilibration at the REST temperature condition *T*_*m*_ (0 ≤ *m* ≤ *R* – 1). The final structures were then used to start REST simulations, ensuring that each replica in each trajectory has a unique initial condition. For all three systems, each replica in each trajectory has been simulated for 100 ns collecting 400 ns per temperature or 4.0 μs of sampling per systems with inhibitor or 3.2 μs without. Following the convergence analysis in the Supporting Information (SI), we concluded that all systems did not exhibit equilibration, allowing us to retain all sampled data. Thus, the total sampling of each system at 310 K is 400 ns. A full analysis of the REST performance and convergence is provided in the SI.

### 4.3 Computation of Structural Probes

Interactions between the inhibitors and the peptide were analyzed by measuring contacts. A contact between the amino acid and inhibitor occurs if a minimal distance between any pair of heavy atoms from the amino acid and inhibitor is less than 4.5 Å. An inhibitor is considered bound to the peptide whenever at least one such contact is present. Based on this definition, we computed the number of binding contacts formed between the peptide and inhibitor. Furthermore, we obtained the probabilities of binding *P*_b_(*j*) of an inhibitor to coreNLS amino acids *j* as well as the overall binding probability *P*_b_. Similarly, we computed the probabilities *P*_b_(*k*) for ligand groups *k* to bind the peptide. We also determined the hydrogen bond formation using VMD.^36^ Here, we used a donor (D)–acceptor (A) cutoff distance of 3.5 Å and a minimum D-H-A angle of 135°. Solvent accessible surface areas (SASA) were computed using VMD.^36^ SASA is further partitioned into apolar SASA (hSASA), which refers to carbon or sulfur atoms, and polar SASA (pSASA) restricted to oxygen or nitrogen atoms. All results represent averages across REST trajectories collected at *T*_0_ = 310 K. Each REST trajectory was considered as a separate sample when calculating standard errors.

### 4.4 Conformational Clustering

To perform density-based conformational clustering of peptide or ligand poses, we used the method of Daura et al.^37^ From each system, a total 10,000 structures selected at a uniform interval across the data sets were collected for clustering. Structural alignment based on the positions of peptide heavy atoms was first performed to minimize the root-mean-square deviation (RMSD) between pairs of peptide structures. After peptide alignment, peptide or ligand poses were clustered using an RMSD-based cutoff of *R*_0_ = 3.7 Å. The populated ligand clusters retained for analysis had to capture at least 1% of all ligand bound poses. To check ligand cluster distributions, we halved the structural data set and repeated clustering. Most of the populated clusters remained unchanged; i.e., the RMSDs between the cluster centroids for the full and halved data sets were less than 2 Å. The populated peptide clusters had to capture at least 15% of the structures. Small errors in the cluster populations of no more than 2% indicate cluster convergence.

## Data Availability

NAMD is available at https://www.ks.uiuc.edu/Research/namd/. VMD is available at https://www.ks.uiuc.edu/Research/vmd/. Initial structures, topology files, REST configuration files, codes for data analysis, and figure data are available at https://github.com/bdelfing/Binding-of-Venezuelan-Equine-Encephalitis-Virus-Inhibitors-to-Importin-Alpha.

## Supporting Information Available

Analysis of REST performance and convergence; analysis of inhibitor bound and unbound states definitions; and additional data on the coreNLS binding to inhibitor groups (PDF)
