# Supplementary material for: Binding of Inhibitors to Nuclear Localization Signal Peptide from Venezuelan Equine Encephalitis Virus Capsid Protein Explored with All-Atom Replica Exchange Molecular Dynamics
Source: ACS Omega. 2024 Sep 16;9(38):40259–68. doi: 10.1021/acsomega.4c06981 (PMC11425950; doi:10.1021/acsomega.4c06981)
Supplement: Supplementary file 1 — ao4c06981_si_001.pdf [file ao4c06981_si_001.pdf]

Supporting Information

Binding of Inhibitors to Nuclear Localization Signal Peptide from Venezuelan Equine Encephalitis Virus Capsid Protein Explored with All-Atom Replica Exchange Molecular Dynamics

Bryan M. Delfing[1], Xavier E. Laracuente[1], Xingyu Luo[1], Audrey Olson[1], William Jeffries[1], Kenneth W. Foreman[2], Mikell Paige[2,3], Kylene Kehn-Hall[4,5], Christopher Lockhart[1], and Dmitri K. Klimov[1*]

[1]School of Systems Biology, George Mason University, Manassas, VA 20110, USA
[2]Department of Chemistry and Biochemistry, George Mason University, Fairfax, VA 22030, USA
[3]Center for Molecular Engineering, George Mason University, Manassas, VA, 20110
[4] Department of Biomedical Sciences and Pathobiology, Virginia-Maryland College of Veterinary Medicine, Virginia Polytechnic Institute and State University, Blacksburg, VA 24061, USA
[5]Center for Emerging, Zoonotic, and Arthropod-borne Pathogens, Virginia Polytechnic Institute and State University, Blacksburg, VA 24061, USA
*E-mail: dklimov@gmu.edu

REST algorithm performance: We tested the technical performance of the REST algorithm following the approach adopted in our previous studies [1,2]. Our REST simulations of inhibitor binding to the coreNLS used R = 10 replicas placed at the temperatures distributed geometrically from T_0 = 310 K to T_{R-1} = 510 K (see Models and Methods). We first qualitatively assessed the random walk of replicas across the temperature range. This behavior is evident in Fig. S1, where replica visits to different temperatures create a random colorful mosaic, suggesting their random walk across temperatures. This color mosaic does not reveal trappings of replicas at any particular temperature.

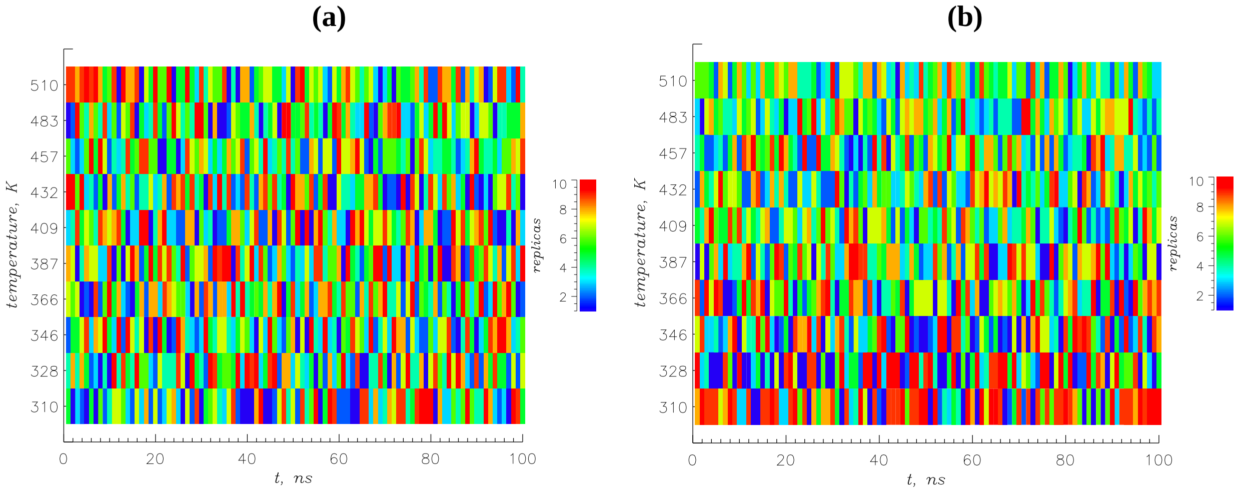

Figure S1 Walks of REST replicas across temperature conditions in one of the trajectories probing binding of I1 (a) or I2 (b) inhibitors to the coreNLS peptide. The color scales show an assignment of replicas to temperatures at the trajectory start. Other REST trajectories as well as ligand-free simulations exhibited similar behavior.

Second, quantitative assessment of the distribution of replicas across temperatures is provided by the replica mixing parameter [3],

$$m(T) = 1 - \frac{\sqrt{\sum_{r=0}^{R-1} t_r^2}}{\sum_{r=0}^{R-1} t_r}, \qquad \text{(S1)}$$

where T is the REST temperature and t_r is the time spent by replica r at T. If $R = 10$ replicas are randomly mixed across the temperature conditions, $m(T)$ acquires the maximum value $m_r = 1 - 1/R^{1/2} = 0.68$. It follows from Fig. S2 that for both REST simulations featuring I1 or I2 inhibitors $m(T)$ approaches m_r. Indeed, the average $m(T)$ across all temperatures are 0.68 and 0.66 for I1 and I2 simulations. For the ligand-free system $R = 8$ and $m_r = 0.65$. For these simulations the average $m(T)$ is equal to m_r. Third, Fig. S3 presents the replica exchange rates $\alpha(T)$. Although there is a gradual increase in $\alpha(T)$ with T, the average exchange rate is 0.39 for both I1 and I2 simulations. For the ligand-free simulations it is 0.32. All these values are close to optimum [4]. Together Figs. S1-S3 demonstrate a good mixing of replicas across temperature conditions as expected from REST formalism.

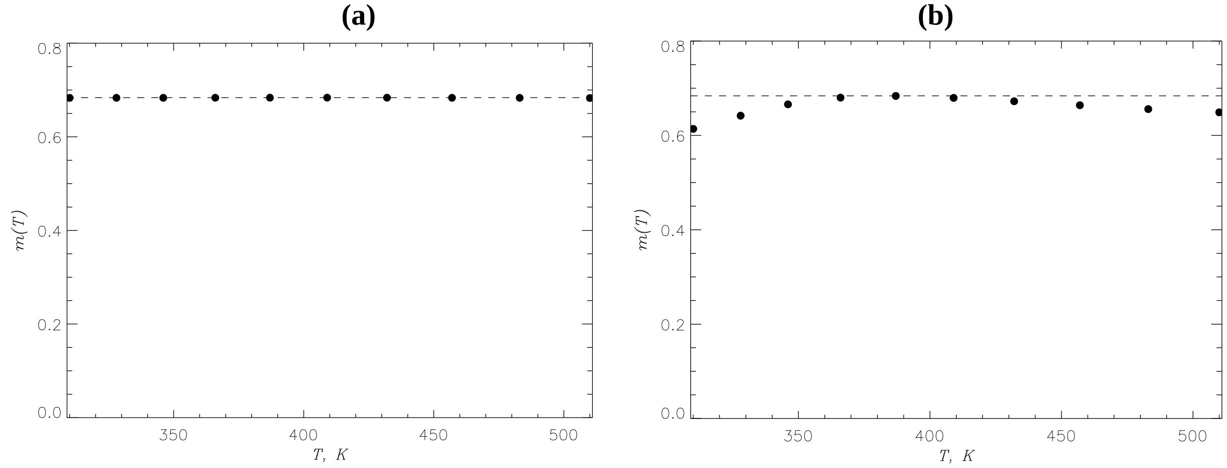

Figure S2 The replica mixing parameter $m(T)$ as a function of REST temperatures T for the simulations probing I1 (a) and I2 (b) binding to the coreNLS peptide (solid circles). The maximum theoretical value $m_r = 0.68$ is marked by a dashed line. The data are averaged across four REST trajectories. Standard errors are too small to show. The plot of $m(T)$ for the ligand-free system is very similar.

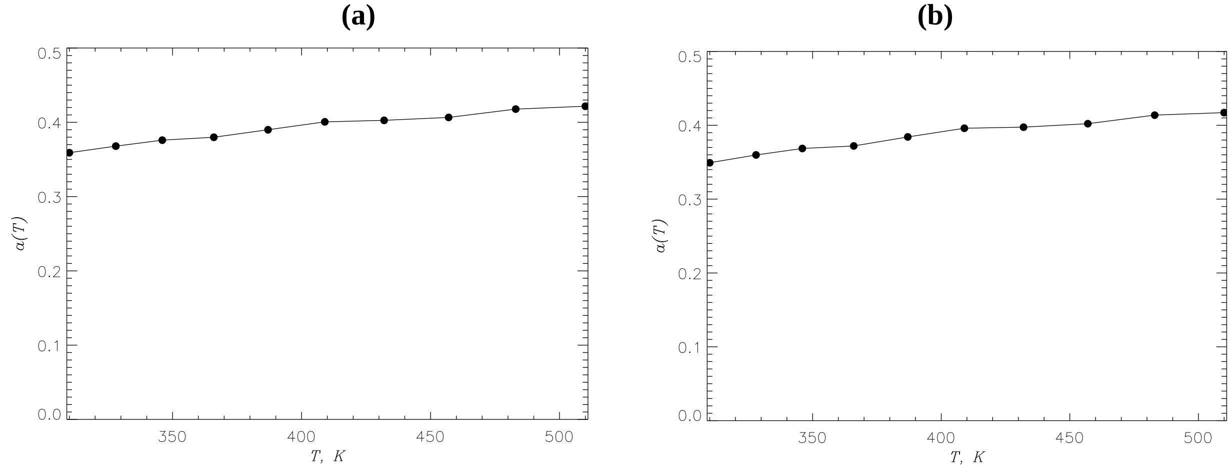

Figure S3 Replica exchange rates $\alpha(T)$ plotted as a function of REST temperatures T for the simulations probing I1 (a) and I2 (b) inhibitor binding to the coreNLS peptide. The data are averaged across four REST trajectories. Standard errors are too small to show. The plot of $\alpha(T)$ for the ligand-free system is very similar.

Convergence of REST sampling: We probed the convergence of REST sampling of the inhibitor binding to the coreNLS in two ways. First, in Fig. S4a,b we plot the numbers of contacts $C(t)$ forming between the

ligand and the coreNLS peptide (see Models and Methods). It is seen that for both I1 and I2 simulations $C(t)$ fluctuates around the baseline over the entire range of 100 ns sampling.

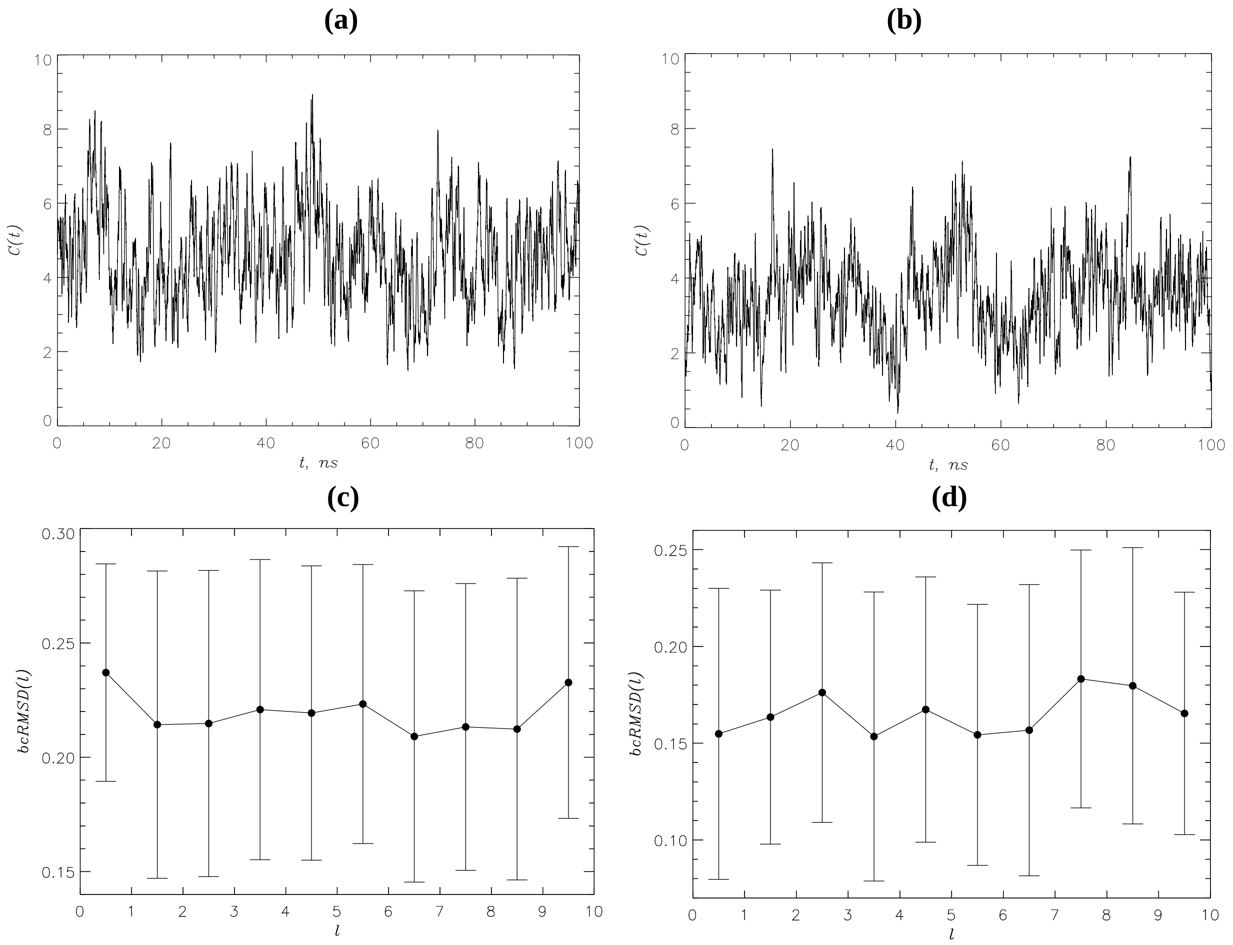

Figure S4 (a,b) The numbers of contacts $C(t)$ forming between the inhibitor I1 (a) or I2 (b) and the coreNLS peptide as a function of REST sampling time t. $C(t)$ are averaged over four REST trajectories and collected at 310 K. The data are smoothed with the sliding window of 200 ps. (c,d) The binding contact root mean squared deviations $bcRMSD(l)$ computed as a function of time windows l for the binding of I1 (c) and I2 (d) to the coreNLS peptide. All plots suggest an absence of equilibration interval in the binding of I1 and I2 to the coreNLS peptide.

To provide a more sensitive verification of REST convergence, we tracked the formation of individual contacts between coreNLS amino acids and ligand groups [1]. To this end, we first computed the contact maps $c(j,k;t,n)$ between the coreNLS amino acids j and inhibitor groups k at time t in a REST trajectory n. Then, the REST timeline was divided into ten 10 ns windows l, and $c(j,k;t,n)$ for a given inhibitor were averaged within a given window l resulting in $C(j,k;l,n)$. Using $C(j,k;l,n)$ we computed the binding contact root mean squared deviation $bcRMSD(l)$

$$bcRMSD\,(l) = \left[\frac{1}{N_{tr}}\sum_n \frac{1}{N_c}\sum_{j,k}\left(C(j,k;l,n) - c^{ref}(j,k;n)\right)^2\right]^{\frac{1}{2}}, \qquad \text{(S2)}$$

where $c^{ref}(j,k;n)$ represents the contact map computed for the initial structure in a REST trajectory n at 310 K, N_{tr} is the number of REST trajectories, N_c is the total number of possible peptide-inhibitor contacts, and

l=0, …, 9. Thus, Eq. (S2) tracks the formation of new or dissociation of existing binding contacts. Fig. S4c,d shows the respective *bcRMSD(l)* for I1 and I2 binding simulations. Consistent with Fig. S4a,b both *bcRMSD*(l) plots do not reveal any ongoing equilibration process. Therefore, we conclude that binding of I1 and I2 necessitates no equilibration intervals. As a result, the entire sampling of 400 ns per system (100 ns per trajectory) at 310K can be used for analysis of I1 and I2 binding to the coreNLS peptide.

To probe the equilibration in the ligand-free system, we first plotted the coreNLS radius of gyration $R_g(t)$ in Fig. S5a. The plot clearly shows that $R_g(t)$ fluctuates around the baseline over the entire length of REST sampling. To provide a more stringent test, we defined the contact root mean squared deviation *pcRMSD*(l), which tracks intrapeptide contacts using the intrapeptide contact maps $c_p(i,j)$, where i and j are indices of the coreNLS amino acids. In all other aspects, the computation of *pcRMSD*(l) followed Eq. (S2). As for I1 and I2 binding simulations, the sampling of the ligand-free coreNLS shows no evidence of equilibration process allowing us to take the entire REST trajectories as equilibrated.

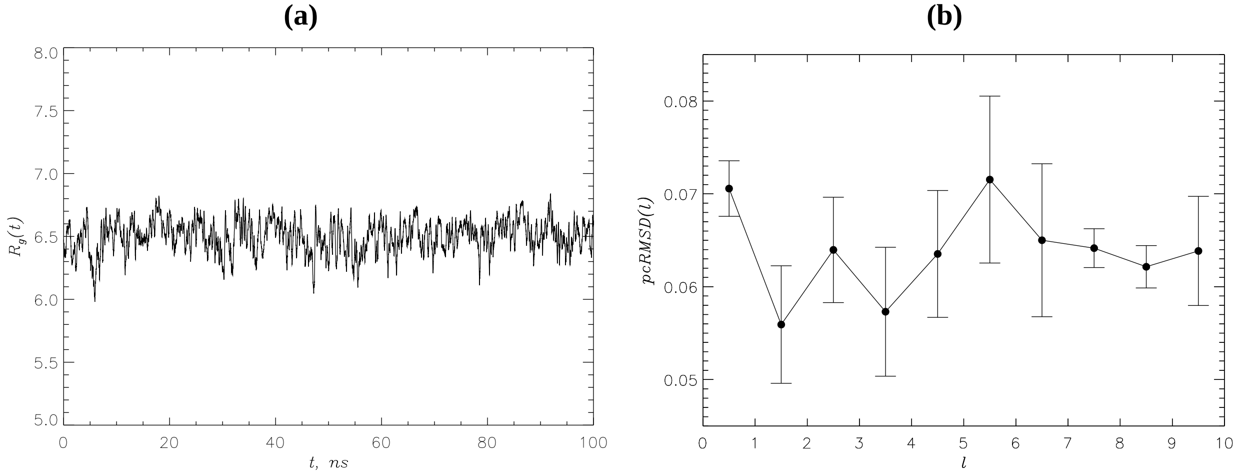

Figure S5 (a) The radius of gyration $R_g(t)$ of the coreNLS peptide in the ligand-free simulations as a function of REST sampling time t. $R_g(t)$ is averaged over four REST trajectories and collected at 310 K. The data are smoothed with the sliding window of 200 ps. (b) The intrapeptide contact root mean squared deviations *pcRMSD*(l) computed as a function of time windows l in the ligand-free simulations. Both panels suggest an absence of equilibration interval in the ligand-free simulations.

Inhibitor unbound state: To utilize Eq. (1) in the main text, we need to verify that the inhibitor forms no interactions with the peptide in the unbound state. To this end, we computed the probability distribution $P(r)$ of finding an inhibitor at the distance r from the peptide. Specifically, r is defined as a minimal separation between peptide and inhibitor heavy atoms. Fig. S6 shows the corresponding free energy $G(r) = -RT\ln P(r)$ for both inhibitors. It is seen that I1 or I2 form a thermodynamically stable bound state at $r \approx 3$ Å, whereas an unbound state is represented by a broad shoulder approximately occurring between 6 Å and 13 Å. A previous study analyzing CHARMM force field suggested that TIP3P water orientation becomes essentially random at $r > 6$ Å from the protein surface [5]. Note that r in [5] is defined as a minimal distance between protein oxygen or nitrogen atoms and water oxygens. Therefore, it represents an upper boundary for the minimal distance between peptide and ligand heavy atoms used by us. Thus, we conclude that the inhibitor unbound state is approximately unaffected by the peptide justifying Eq. (1) in the main text.

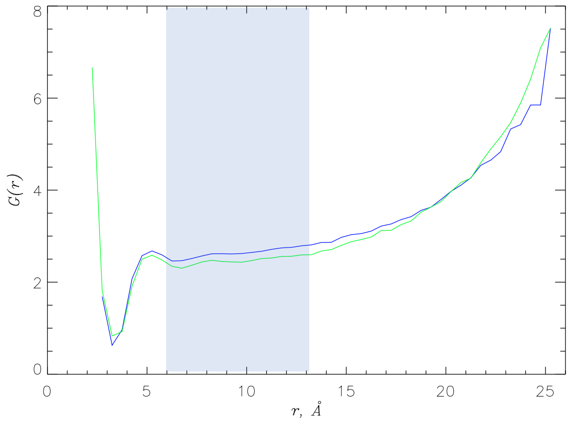

Figure S6 The inhibitor free energy $G(r)$ as a function of a minimal distance r between peptide and ligand heavy atoms. Data in blue and green are collected for I1 and I2. The shaded region approximately corresponds to the unbound state.

Impact of bound state definition on binding free energy: In Model and Methods we assumed that an inhibitor is bound if it forms at least one contact with the coreNLS peptide. A contact occurs when a distance between any pair of heavy atoms from the peptide and inhibitor is less than 4.5 Å. The corresponding free energies ΔG_b for I1 and I2 binding to the coreNLS and impα are shown in Table S1 ("original definition"). Previous studies have shown that ΔG_b may be affected by the bound state definition [6]. To test the robustness of ΔG_b, we used two alternative contact definitions. In the first, a contact occurs if the distance between the centers of mass of an amino acid and any ligand group is less than 6.5 Å. The second definition is similar to the first except that it considers the center of mass of a side chain rather than an entire amino acid. The resulting free energies ΔG_b are shown in Table S1 ("alternative definitions"). It is seen that ΔG_b depends weakly on the specific definition of the bound state.

Table S1 Binding free energies ΔG_b in kcal/mol computed using different definitions of bound state.

	Original definition[a]		First alternative definition[a]		Second alternative definition[a]	
	coreNLS	impα	coreNLS	impα	coreNLS	impα
I1	-2.4	-5.2	-2.3	-4.9	-2.4	-5.2
I2	-2.0	-5.5	-1.9	-5.2	-2.0	-5.5

[a] see text above for the specific bound state definitions.

It is important to comment on the definition of inhibitor binding to impα. Because we study the coreNLS peptide fragment of the NLS sequence, we consider inhibitor binding to all impα amino acids forming native contacts with the coreNLS peptide in 3VE6 structure. Nonetheless, an inhibitor may interact with impα amino acids beyond the coreNLS binding site. Consequently, the computed free energies of inhibitor binding to impα may represent the upper bounds and underestimate the actual affinities to impα. However, this caveat does not impact our central conclusion that both inhibitors bind with stronger affinities to impα than to the coreNLS.

Binding of inhibitor groups to the coreNLS peptide: To gain better insight into binding mechanism, we examined the interactions between inhibitor groups (see Fig. 1a) and the coreNLS peptide. Fig. S7 shows the probabilities $P_b(k)$ of peptide binding to the ligand group k. The implications are discussed in the main text.

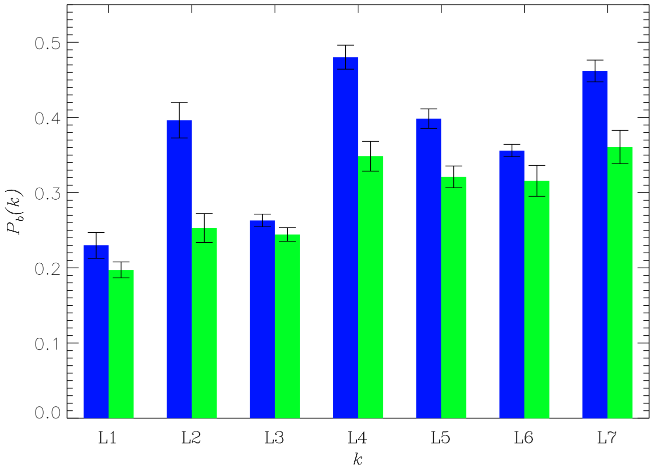

Figure S7 Probabilities $P_b(k)$ of peptide binding to the ligand group k. Data in blue and green are collected for I1 and I2.

References

[1] Delfing, B. M., Laracuente, X. E., Olson, A., Foreman, K. W., Paige, M., Kehn-Hall, K., Lockhart, C., and Klimov, D. K. (2023) Binding of Viral Nuclear Localization Signal Peptides to Importin-α Nuclear Transport Protein. *Biophys. J.* **122**, 3476-3488.

[2] Delfing, B. M., Olson, A., Laracuente, X., Foreman, K. W., Paige, M., Kehn-Hall, K., Lockhart, C., and Klimov, D. K. (2023) Binding of Venezuelan Equine Encephalitis Virus Inhibitors to Importin-α Receptors Explored with All-Atom Replica Exchange Molecular Dynamics. *J. Phys. Chem.* B **127**, 3175–3186.

[3] Han, M. and Hansmann, U. H. E. (2011) Replica exchange molecular dynamics of the thermodynamics of fibril growth of Alzheimer's Aβ42 peptide. *J. Chem. Phys.* **135**, 065101.

[4] Denschlag, R., Lingenheil, M., and Tavan, P. (2009) Optimal temperature ladders in replica exchange simulations. *Chem. Phys. Lett.* **473**, 193-195.

[5] Qiao, B., Jiménez-Ángeles, F., Nguyen, T. D., and de la Cruz, M. O. (2019) Water follows polar and nonpolar protein surface domains. *Proc. Natl. Acad. Sci. USA* **116**, 19274–19281.

[6] Gilson, M. K., Given, J. A., Bush, B. L., and McCammon, J. A. (1997) The statistical-thermodynamic basis for computation of binding affinities: A critical review. *Biophys J.* **72**, 1047-1069.
